# David E. Bernard

**Published:** 1920-09

**Authors:** 


### Obituary.

### DAVID E. BERNARD, M.R.C.S., L.S.A., L.R.C.P.

Another of the older generation of medical practitioners of this city has passed away, and in the death of David Edward Bernard the profession has lost a man distinguished in his own profession and of exceptional brilliance in many other sciences and arts.

He was a man of fine talents, and had he chosen might have excelled in any branch of medicine, surgery, ophthalmology or midwifery. The last was his speciality, if one can say he had one, and at this he was pre-eminent : many are the patients and medical confrères who in the old days had cause to thank him for his timely help. He was always at the service of anyone, and his knowledge and time were most ungrudgingly given. No one knows more than myself, a close friend for over 45 years, what a kind and sympathetic nature he had and how gentle and painstaking he was in illness. His manner was often brusque, but under it all he had a heart of gold, and there are many who owe a debt of gratitude to him for good advice and timely aid rendered in a moment of stress without a thought for himself.

His life in practice was a strenuous one, and he retired from the activities of his profession about ten years ago ; but he was never idle, and had many hobbies to occupy his spare moments. He was passionately fond of music, and helped many a pupil in their studies. He was an exacting master ; but the time was well spent, as many could testify in their after-life. He could converse well on any subject, and it was easy to see his knowledge was not purely theoretical ; he never took up a subject without thoroughly mastering its every detail. Microscopy, music, art, clocks and watches all came the same to him, and he was an authority on all. He was an ardent sportsman, and in early life was a constant rider to hounds, whilst cricket he imbibed from his cradle, and he was one of the oldest subscribing members of the Gloucestershire Club. He was also a welcome member at all athletic meetings, where he was official timekeeper up to a few years ago. For the last few years of his life he was a great sufferer, but was most patient to the end, when he was thankful to be called home. He died on the 9th of July, aged 78 years, and his body was cremated, as he desired, at Golder's Green.

C. K. C. Herapath.

We are indebted to Dr. W. Hetling for some further notes :—

It is now over fifty years since David Edward Bernard became house pupil at the Bristol Royal Infirmary and the writer became an ordinary pupil there, and thus became acquainted with Bernard, and being considerably older than the general run of pupils, attached himself to him for reading purposes. He was reading for his final and pass, I, of course, as a neophyte and beginner, and he nearly drove me to despair by his exact and thorough knowledge of his work. He chose obstetrics as his more special study, and obtained permission to put up a notice in the Out-patients' Room that notes for such attendance could be obtained free by applying to him. As a matter of fact, he got plenty of applications, thus completing his extensive and most thorough acquaintance with this subject, and soon numbered his thousand cases.

Having obtained his M.R.C.S. and L.S.A., he started practice in the east of Bristol, and applied for the post of Medical Officer

14

to the Eastville Poor Law establishment, to obtain which he took the L.R.C.P. of Edinburgh, and remained as their valued and respected officer until his retirement upon pension. Scarcely, however, was it a period of peace, for his incessant and Homeric differences with the clerk became notorious, and occasionally even the Guardians were made acquainted with Bernard's opinions "very forcibly." Subsequently he obtained plenty of practice, especially in midwifery, where his services became very widely known and valued.

But apart from medicine he was a very many-sided man, and took up optics, music, whist and bridge, and whatever he touched he mastered — no mere superficial acquaintance satisfied him. For years he played the French-horn in George Riseley's orchestra, which is to say that he "knew the horn," and it was only the loss of his front teeth which forced him to abandon it, which he never ceased to regret. For the last two years of his life he suffered much pain, borne with exemplary patience.

